# Exhausted CD4^+^ T Cells during Malaria Exhibit Reduced mTORc1 Activity Correlated with Loss of T-bet Expression

**DOI:** 10.4049/jimmunol.2000450

**Published:** 2020-08-17

**Authors:** Ana Villegas-Mendez, Garima Khandelwal, Lucy M. McGowan, Rebecca S. Dookie, Michael J. Haley, Charlotte George, David Sims, Graham M. Lord, Linda V. Sinclair, Richard G. Jenner, Kevin N. Couper

**Affiliations:** * The Lydia Becker Institute of Immunology and Inflammation, Faculty of Biology, Medicine and Health, University of Manchester, Manchester M13 9PT, United Kingdom;; † University College London Cancer Institute, University College London, London WC1E 6DD, United Kingdom;; ‡ Department of Physiology, Pharmacology and Neuroscience, Faculty of Life Sciences, University of Bristol, Bristol BS8 1TD, United Kingdom;; § Oxford Biomedical Data Science Training Programme, Medical Research Council Wetherall Institute of Molecular Medicine Centre for Computational Biology, Medical Research Council Wetherall Institute of Molecular Medicine, University of Oxford, John Radcliffe Hospital, Oxford OX3 9DS, United Kingdom; and; ¶ Division of Cell Signaling and Immunology, School of Life Sciences, University of Dundee, Dundee DD1 5EH, United Kingdom

## Abstract

CD4^+^ T cell functional inhibition (exhaustion) is a hallmark of malaria and correlates with impaired parasite control and infection chronicity. However, the mechanisms of CD4^+^ T cell exhaustion are still poorly understood. In this study, we show that Ag-experienced (*Ag-exp*) CD4^+^ T cell exhaustion during *Plasmodium yoelii* nonlethal infection occurs alongside the reduction in mammalian target of rapamycin (mTOR) activity and restriction in CD4^+^ T cell glycolytic capacity. We demonstrate that the loss of glycolytic metabolism and mTOR activity within the exhausted *Ag-exp*CD4^+^ T cell population during infection coincides with reduction in T-bet expression. T-bet was found to directly bind to and control the transcription of various mTOR and metabolism-related genes within effector CD4^+^ T cells. Consistent with this, *Ag-exp*Th1 cells exhibited significantly higher and sustained mTOR activity than effector T-bet– (non-Th1) *Ag-exp*T cells throughout the course of malaria. We identified mTOR to be redundant for sustaining T-bet expression in activated Th1 cells, whereas mTOR was necessary but not sufficient for maintaining IFN-γ production by Th1 cells. Immunotherapy targeting PD-1, CTLA-4, and IL-27 blocked CD4^+^ T cell exhaustion during malaria infection and was associated with elevated T-bet expression and a concomitant increased CD4^+^ T cell glycolytic metabolism. Collectively, our data suggest that mTOR activity is linked to T-bet in *Ag-exp*CD4^+^ T cells but that reduction in mTOR activity may not directly underpin *Ag-exp*Th1 cell loss and exhaustion during malaria infection. These data have implications for therapeutic reactivation of exhausted CD4^+^ T cells during malaria infection and other chronic conditions.

## Key Points

- CD4^+^ T cell exhaustion during malaria is associated with reduced mTOR activity.
- mTOR activity and glycolysis in CD4^+^ T cells are correlated with T-bet expression.
- Blocking T cell exhaustion increases T-bet expression and elevates glycolysis.

## Introduction

Malaria, caused by infection with *Plasmodium* spp. parasites, continues to be a major health and economic burden in many parts of the world (1, 2). CD4^+^ T cells orchestrate protective immunity to blood-stage malaria by activating macrophages to kill the parasite and helping B cells produce Ab (3–5). Studies of human malaria and experimental murine models of infection have, however, shown that effector CD4^+^ T cells become hyporesponsive (or exhausted) during the course of malaria, with loss of proliferative capacity, repressed cytokine production, and reduced ability to help macrophages and B cells (3, 5, 6). The loss of effector CD4^+^ T cell function during malaria infection directly correlates with impaired parasite control and the establishment of chronic malarial infections (3).

It is increasingly understood that T cell activation, differentiation, and effector function are all intrinsically governed by cellular metabolic programs (7–10). TCR signals, IL-2, and costimulatory pathways converge to activate mammalian target of rapamycin complex 1 (mTORc1), which is a critical metabolic hub that promotes anabolic metabolic programs, such as glycolysis and amino acid metabolism, necessary for T cell proliferation and de novo macromolecule generation (7–10). Thus, mTORc1 is nonredundantly required for generation of effector T cell subsets, including Th1 cells, Th17 cells, and CTLs, during various different inflammatory conditions and infections and for controlling effector T cell functions, such as IFN-γ and granzyme B production (8, 9, 11). Notably, there is evidence that mTORc1 and anabolic metabolic programs are repressed in tumor-infiltrating effector T cells and in exhausted effector T cells during chronic viral infections (12–14). The importance of mTOR in T cell activation during malaria infection and whether alterations in metabolic programming also underlie CD4^+^ T cell functional exhaustion during malaria infection has not been examined.

Various coinhibitory pathways and regulatory cytokines have been shown to play roles in inhibiting T cell proliferation and effector function during malaria infection (15–17). Abrogation of cell surface regulatory receptor activities, including blockade of PD1, CTLA-4, LAG-3, Tim3, and BTLA, have been shown to improve CD4^+^ T cell and/or CD8^+^ T cell responses and enhance parasite control during different murine *Plasmodium* spp. infections (15, 18–22). Moreover, PD-1, CTLA-4, LAG-3, and Tim-3 have been suggested to contribute to T cell immunosuppression during human *Plasmodium falciparum* infection (15, 21–24). We have also previously shown that IL-27 plays a nonredundant dominant role in limiting the magnitude of Th1 cell responses during murine malaria (25, 26). Consequently, targeting regulatory pathways has been proposed as a therapeutic strategy during malaria infection (15, 27). Although we still have limited mechanistic understanding of how regulatory pathways suppress effector T cell responses during malaria infection, PD-1 and CTLA-4 have been shown to inhibit mTOR activity in T cells during in vitro stimulation experiments (28, 29).

In this study, we have examined whether CD4^+^ T cell exhaustion during malaria infection is orchestrated through changes in mTOR-dependent cellular metabolism. We demonstrate that lowered mTOR activity in effector CD4^+^ T cells during the course of *Plasmodium yoelii* nonlethal (*Py*NL) infection correlates with the temporal loss of T-bet expression. T-bet directly bound to and activated the expression of various mTOR-activating and metabolic genes, and Th1 cells exhibited high levels of sustained mTOR activity compared with T-bet–deficient effector CD4^+^ T cells throughout the course of infection. Notably, immunotherapy to block T cell exhaustion during malaria infection raised CD4^+^ T cell glycolytic metabolism associated with an increase in T-bet expression. Our results define T-bet as a potentially important controller of mTORc1 activity in effector T cells and have implications for understanding the mechanisms of Th1 cell exhaustion and functional repression during malaria infection and other chronic infections.

## Materials and Methods

### Ethics

Animal work was approved by the University of Manchester Animal Procedures and Ethics Committee and was performed in accordance with the U.K. Home Office Animals (Scientific Procedures) Act 1986 (project licenses 70/7293 and P8829D3B4).

### Mice and parasites

C57BL/6 mice were purchased from Charles River Laboratories, Margate, U.K. and were maintained at the University of Manchester. IL-27R–deficient [WSX-1^−/−^ mice (30)] were bred and maintained at the University of Manchester. mTOR^flox/flox^ mice (31) obtained from The Jackson Laboratory, were crossed with CD4-Cre mice (32) to generate T cell–specific mTOR-deficient mice (CD4-Cre^+/−^ mTOR^flox/flox^) and littermate control mice (CD4-Cre^−/−^ mTOR^flox/flox^). All mice were housed in individually ventilated cages (transgenic mice and controls were cohoused within cages). Sex-matched 6–10-wk-old age-matched mice were used in separate experiments.

Cryopreserved *Py*NL or *Plasmodium berghei* NK65-GFP parasites (33) were thawed and passaged once through C57BL/6 mice. Experimental mice were subsequently infected i.v. with 1 × 10^4^ parasitized RBCs (pRBCs) by injection into the tail vein. In some experiments, randomized mice were injected i.p. with 250 μg of anti-mouse PD-L1 (clone 10F.9G2) and anti-mouse CTLA-4 (clone UC10-4F10-11) every 2 d, from day 3 postinfection. All Abs were from Bio X Cell (West Lebanon, NH). Peripheral parasite burdens were monitored every second day of infection by microscopic examination of Giemsa-stained thin blood smears.

### Flow cytometry

Spleens were collected from naive and malaria-infected mice. Single-cell suspensions were prepared by homogenizing tissue through a 70-μm cell strainer (BD Biosciences). RBCs were lysed (RBC Lysing Buffer; BD Biosciences), and samples were washed in FACS buffer (HBSS with 2% FCS) and resuspended in RPMI 1640 supplemented with 10% FCS. Live/dead cell counting and absolute cell numbers were calculated by trypan blue exclusion (Sigma-Aldrich) using a C-Chip (NanoEntek, Pleasanton, CA). For all staining protocols, 4 × 10^6^ cells per sample were washed with PBS and stained with LIVE/DEAD Fixable Blue Dead Cell Stain Kit for UV excitation (Life Technologies). Samples were then surface stained with anti-mouse Abs against CD4 (RM4-5), CD8 (53-6.7), ICOS (C398.4A), KLRG-1 (2F1), CD25 (PC61), CD98 (4F2), CD71 (R17217), PD-1 (29F.1A12), CD11a (M17/4), CD49d (R1-2), CD44 (Im7), CD62L (MEL-14), and CXCR5 (1.138D7). For intracellular staining, surface-stained cells were washed in FACS buffer and permeabilized with Foxp3 fixation/permeabilization buffers (eBioscience, Thermo Fisher Scientific) for 30 min. The cells were then stained with anti-mouse Abs against Ki-67 (SolA15), T-bet (4B10), CTLA-4 (UC10-4B9), GATA3 (TWAJ), Foxp3 (FJK-165), Bcl-6 (7D1), Glut-1 (FAB1418R), and c-Myc (D84C12).

For intracellular staining of phosphorylated proteins, an alternative protocol was used (as described in Ref. 25). Briefly, splenocytes were kept on ice and immediately fixed for 15 min on ice by addition of an equal volume of 4% paraformaldehyde. Cells were permeabilized with 90% ice-cold methanol at −20°C overnight and then stained for CD4, CD49d, CD11a, T-bet, pS6 (Ser^235^, Ser^236^) (cupk43k), p4EBP1 (Thr^36^, Thr^45^) (V3NTY24), and pAKT (Ser^473^) (700392).

Intracellular IFN-γ and TNF production was assessed ex vivo on whole splenocytes. For this, 1 × 10^6^ live cells were incubated in RPMI 1640 medium supplemented with 10% FCS, 200 ng/ml PMA (Sigma-Aldrich) and 1 μg/ml ionomycin (Sigma-Aldrich) in the presence of brefeldin A (1∶1000; eBioscience, Thermo Fisher Scientific) for 4 h at 37°C, 5% CO_2_. Samples were surfaced stained first, followed by intracellular staining as described above, using anti-mouse Abs against IFN-γ (XMG1.2) and TNF (MP6-XT22) for 30 min. All Abs were from eBioscience Thermo Fisher Scientific, BioLegend or Novus Biologicals. Fluorescence-minus-one controls were performed to validate Ab staining. Data acquisition was performed using a Fortessa instrument (BD Systems), and analysis was performed using FlowJo software (Tree Star, Ashland, OR).

### Quantification of cytokine secretion by CD4^+^ T cells

Splenic CD4^+^ T cells from naive and malaria-infected mice (day 5, 9, and 15 of infection) were positively selected by magnetic cell sorting as described above. A total of 2 × 10^5^ cells were incubated in RPMI 1640 medium supplemented with 10% FCS, 200 ng/ml PMA (Sigma-Aldrich) and 1 μg/ml ionomycin (Sigma-Aldrich) on 96-well plates for 4 h at 37°C, 5% CO_2_. Cell supernatants were stored at −80°C until further use. The concentrations of IL-2, IFN-γ, TNF, and IL-10 in the supernatants were measured by a Cytometric Bead Array Mouse Th1/Th2/Th17 Cytokine Kit (BD Biosciences), following the manufacturer’s instructions.

### Inhibition of mTOR activity on in vitro–activated Th1 cells

Splenic CD4^+^ T cells from naive mice were positively selected by magnetic cell sorting using anti-CD4 microbeads (Miltenyi Biotech), according to the manufacturer’s guidelines. Cells were seeded at a density of 1 × 10^6^ cells in a 24-well plate, and activated with 2 μg/ml anti-CD3 (BD Biosciences) and 2 μg/ml anti-CD28 (eBioscience). Cells were grown for 3 d under Th1-polarizing conditions (10 ng/ml rIL-12 [R&D Systems]) 10 μg/ml anti–IL-4 (clone 11B11; eBioscience, Thermo Fisher Scientific), split, and further grown until day 4 or 5 under Th1-polarizing conditions and 5 ng/ml rIL-2 (R&D systems). mTOR signaling was blocked by addition of 250 nM Torin (Tocris Bioscience, Bio-Techne) or PBS vehicle control either from day 0 or 3. mTOR signaling, T-bet expression, and IFN-γ production in CD4^+^ CD44^+^ T cells was measured by flow cytometry, as described above.

### Inhibition of mTOR activity on infection-derived splenocytes

Splenocytes from naive and malaria-infected mice were prepared as described above and incubated for 40 min at 37°C, 5% CO_2_ at a density of 6 × 10^6^ cells on 48-well plate with or without anti-CD3/anti-CD28, as described above. mTOR activity was inhibited by addition of 250 nM Torin. mTOR signaling and T-bet expression in CD4^+^ CD49d^+^ CD11a^+^ T cells was measured by flow cytometry, following the intracellular phosphorylation protocol described above.

### Extracellular flux metabolic assay

Splenic CD4^+^ T cells from naive and malaria-infected mice (day 5 and 15 of infection) were positively selected by magnetic cell sorting as described above. Cells were plated at a density of 200,000 cells per well on Cell-Tak–coated XFe96 Cell Culture Microplates (Agilent Technologies) in assay media (nonbuffered RPMI 1640 containing 10 mM glucose, 2 mM l-glutamine, and 1 mM sodium pyruvate). Oxygen consumption rates (OCR) and extracellular acidification rates (ECAR) were measured in assay media under basal conditions and in response to mitochondrial inhibitors, 2 μM oligomycin plus 2 μM carbonyl cyanide p-trifluoromethoxyphenylhydrazone and 100 nM rotenone plus 1 μM antimycin A (Sigma-Aldrich), and the glycolysis inhibitor 2-deoxy-d-glucose (50 mM) on the Seahorse XF96 Extracellular Flux Analyzer (Agilent Technologies).

### Image stream

In vitro–activated CD4^+^ T cells were surface stained for 25 min at 4°C with CD4 (RM4-5). For intracellular staining, cells were incubated with Foxp3 fixation/permeabilization buffer for 30 min at 4°C. Cells were then stained with T -bet (4B10) for 30 min at 4°C, before resuspending in PBS containing DAPI. Dead cells were excluded using fixable viability dye (Invitrogen). Cells were analyzed with an ImageStream X Mark II (Amnis), and data were analyzed with IDEAS (Amnis).

### Bioinformatics analysis

RNA-sequencing (RNA-seq) data for wild-type (WT) and T-bet knockout (KO) Th1 cells were downloaded for three different studies from the Gene Expression Omnibus (Ref. 34, GSE40463; Ref. 35, GSE38808; Ref. 36, GSE48138). The data were aligned to the mm10 genome assembly with a splice junctions file generated using Ensembl 81 using STAR (37). RNA abundance was quantified using featureCounts (38) in R, using the Ensembl 97 gene transfer format file. Differential expression analysis was performed between WT and T-bet KO groups with DESeq2 (39) in R (R Core Team. 2017. R: A language and environment for statistical computing. R Foundation for Statistical Computing, Vienna, Austria. Available at https://www.R-project.org/). Significantly differentially expressed genes (adjusted *p* value <0.01) were sorted on the basis of log_2_ fold change values plotted as a heatmap using pheatmap in R (R. Kolde. 2019. pheatmap: Pretty Heatmaps. R package version 1.0.12. Available at https://CRAN.R-project.org/package=pheatmap). Gene enrichment analysis was performed on the significantly upregulated genes using the gProfileR package (40) in R. Sets of genes associated with enhancers and superenhancers were taken from Hertweck et al. (41), and gene enrichment analysis was performed with the gProfileR package in R. T-bet chromatin immunoprecipitation sequencing data were taken from GSE62486 (41) and realigned to *Mus musculus* 10 with bowtie2 (–very-sensitive parameters) (42). Replicate RNA-seq bam files were merged, and bigWig files were generated with the bamCoverage function (–normalizeUsing RPGC–binSize 10 parameter) from deepTools (43) for visualization in the UCSC Genome Browser.

### Statistical analysis

Except for RNA-seq data, all statistical analyses were performed using GraphPad Prism (GraphPad Software). Data were checked for normality using the Shapiro–Wilk test. For normally distributed data, statistical significance for two-group comparisons was determined using the *t* test; nonparametric data were analyzed using the Mann–Whitney *U* test. Statistical significance on three or more group comparisons was determined using one-way or two-way ANOVA with Tukey post hoc analysis (parametric data) or the Kruskal–Wallis test with a Dunn post hoc test (nonparametric data). Results were considered significantly different when the *p* value was <0.05.

## Results

### mTOR is required for development of Ag-experienced CD4^+^ T cell responses during *Py*NL infection

**FIGURE 1.**
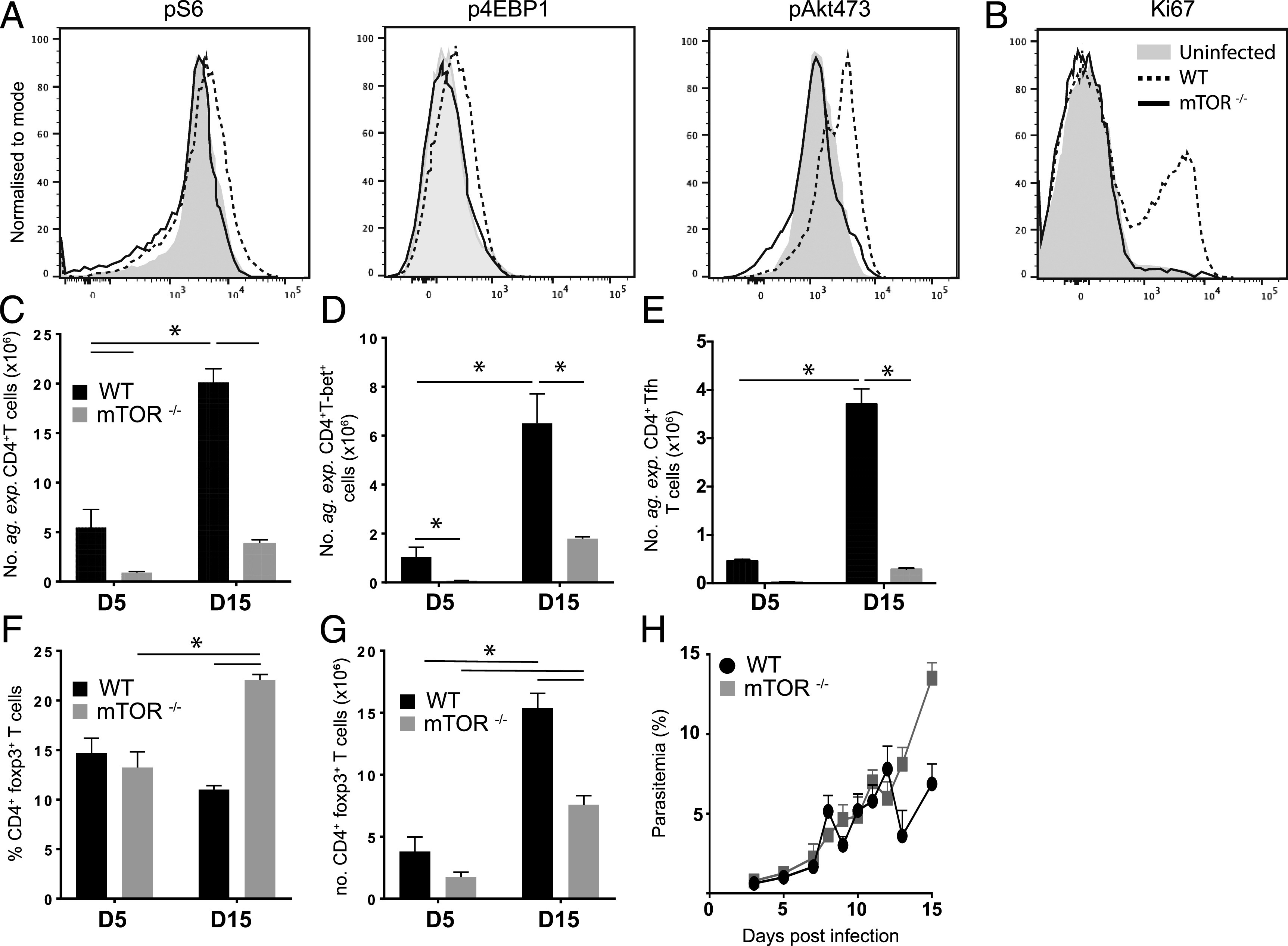
mTOR is required for development of an effector CD4^+^ T cell response during malaria infection. T cell–specific mTOR KO and littermate (WT) mice were infected i.v. with 1 × 10^4^ *Py*NL pRBCs. (**A**) Representative histograms showing the levels of pS6, p4EBP1, and pAKT^473^ in splenic CD4^+^ T cells on day 5 postinfection compared with naive CD4^+^ T cells from uninfected mice. (**B**) Representative histogram showing the level of Ki67 in splenic CD4^+^ T cells on day 5 postinfection, compared with naive CD4^+^ T cells from uninfected mice. (**C**) The numbers of *Ag-exp*CD4^+^ T cells (CD11a^+^CD49d^+^) in the spleen of T cell–specific mTOR KO and WT mice on days 5 and 15 postinfection. (**D** and **E**) The numbers of splenic (D) *Ag-exp*CD4^+^ T-bet^+^ cells and (E) *Ag-exp*CD4^+^ Tfh (PD-1^+^CXCR5^+^) cells on days 5 and 15 postinfection. (**F** and **G**) The (F) proportions and (G) numbers of CD4^+^ Foxp3^+^ cells on days 5 and 15 postinfection. (**H**) Peripheral parasitemia as measured by Giemsa thin blood smear. Results are mean ± SEM of the group (*n* = 3–4) from one of two or three independent experiments. *p < 0.05 between defined groups by *t* test or two-way ANOVA with Tukey post hoc test.

The specific requirement of T cell–intrinsic mTOR expression and activation for generation of T cell responses during malaria infection is unknown. Thus, to investigate this we infected T cell–specific mTOR^Δ/Δ^ mice (CD4-cre^+/−^ X mTOR^flox/flox^) with *Py*NL. Phosphorylation of S6 and 4EBP1 (associated with mTORc1 activity) and AKT^473^ (associated with mTORc2 activity) was observed on day 5 of infection in CD4^+^ T cells from WT littermates but not from mTOR^Δ/Δ^ animals, indicating that the mTORc1 and mTORc2 pathways were activated in WT CD4^+^ T cells during the initiation of the immune response during malaria infection (Fig. 1A). mTOR-deficient CD4^+^ T cells failed to upregulate Ki67 expression during *Py*NL infection, indicative of a failure to proliferate (Fig. 1B). Consequently, the number of splenic Ag-experienced (*Ag-exp*) CD11a^+^CD49d^+^ CD4^+^ T cells, which contain parasite-specific CD4^+^ T cell populations (22, 44), was significantly attenuated in mice with a CD4 T cell–specific mTOR deficiency on days 5 and 15 of infection (Fig. 1C). The *Ag-exp*CD4^+^ T cells that did form in T cell–specific mTOR^Δ/Δ^ mice also showed defective signs of activation, including reduced expression of ICOS, CXCR3, and the coinhibitory molecules PD-1 and CTLA-4 (results not shown). Combined, this led to the abrogated expansion of effector Th1 (T-bet^+^) and T follicular helper (Tfh; CXCR5^+^PD-1^+^) subsets in T cell mTOR^Δ/Δ^ mice during the course of *Py*NL infection (Fig. 1D, 1E). Interestingly, although the proportions of Foxp3^+^ regulatory T cells were increased (Fig. 1F), there was still a significant reduction in the overall number of Foxp3^+^ regulatory T cells in T cell–specific mTOR^Δ/Δ^ mice at the later stage of infection (Fig. 1G). Consistent with these data, parasite control was reduced in T cell–specific mTOR^Δ/Δ^ mice at the later stages of *Py*NL infection (Fig. 1H). Overall, these data highlight the critical requirement for mTOR in CD4^+^ T cells for the development of an effector *Ag-exp*CD4^+^ T cell response during *Py*NL infection.

### mTOR activation is not sustained in *Ag-exp*CD4^+^ T cells, which become functionally impaired during the course of *Py*NL infection

**FIGURE 2.**
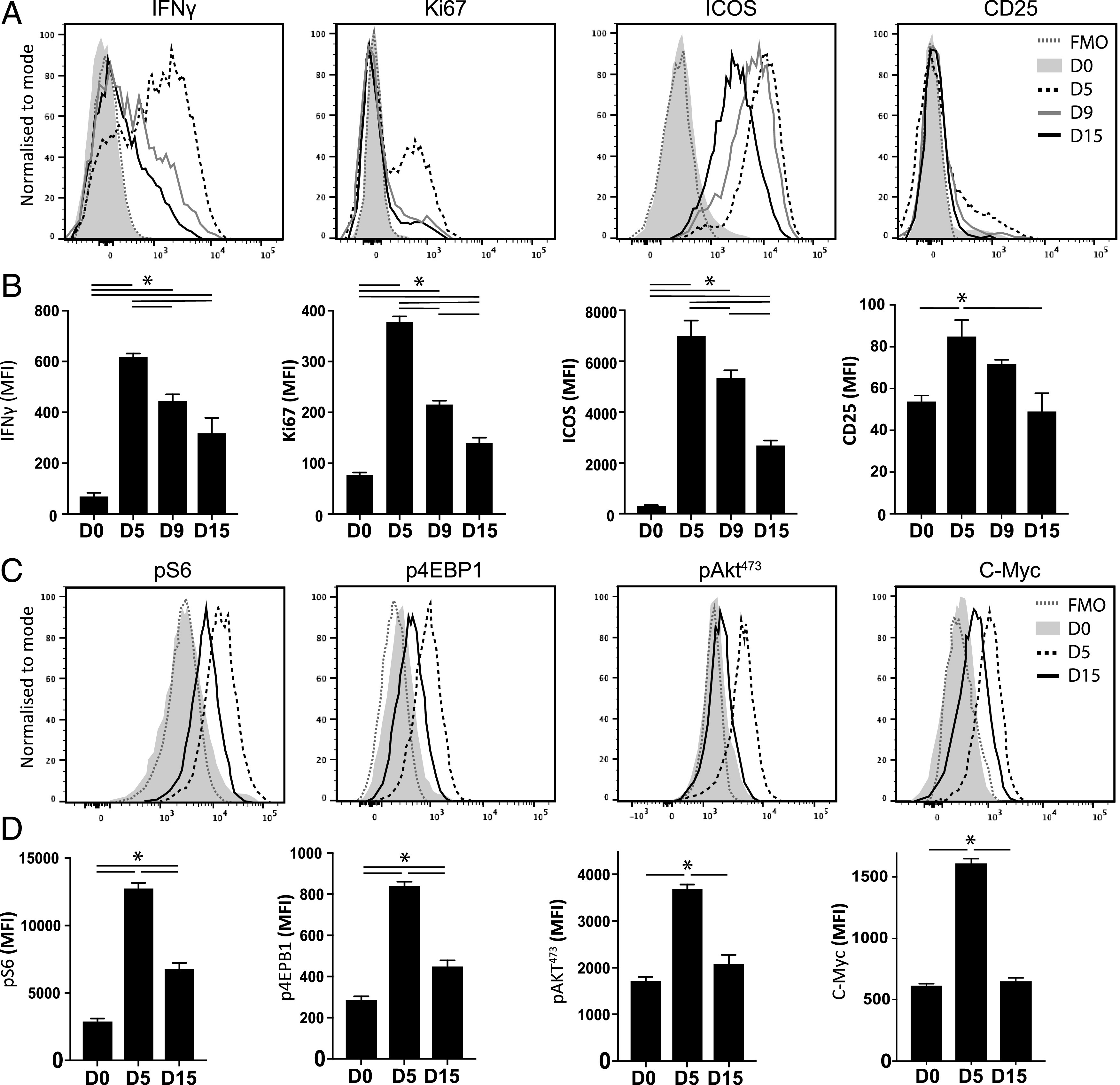
mTOR activity declines in *Ag-exp*CD4^+^ T cells concomitant with loss of T cell functionality during *Py*NL infection. C57BL/6 mice were infected i.v. with 1 × 10^4^ *Py*NL pRBCs. (**A**) Representative histograms and (**B**) calculated mean fluorescence intensity (MFI) (mean of the group) for IFN-γ, Ki67, ICOS, and CD25 in splenic *Ag-exp*CD4^+^ T cells during the course of infection compared with naive CD4^+^ T cells from uninfected mice (day 0). (**C**) Representative histograms and (**D**) calculated MFI (mean of the group) for pS6, p4EBP, pAKT^473^, and c-Myc in splenic *Ag-exp*CD4^+^ T cells during the course of infection compared with naive CD4^+^ T cells from uninfected mice (day 0). Results are the mean ± SEM of the group (*n* = 4) and are from one of three or four independent experiments. *p < 0.05 between defined groups by one-way ANOVA with Tukey post hoc test.

Having verified the importance of T cell–intrinsic mTOR activity in development of effector T cell responses during malaria infection, we examined the dynamics of mTOR activity during the onset of T cell exhaustion. *Ag-exp*CD4^+^ T cells rapidly became exhausted during *Py*NL infection in WT mice. On day 5 of infection, *Ag-exp*CD4^+^ T cells were highly proliferative and exhibited a proinflammatory phenotype, as defined by production of IFN-γ and expression of Ki67, ICOS, and CD25 (Fig. 2A, 2B). However, by day 9 postinfection, *Ag-exp*CD4^+^ T cells had a reduced capacity to produce IFN-γ compared with *Ag-exp*CD4^+^ T cells on day 5 of infection and exhibited low expression of Ki67, indicating minimal active proliferation (Fig. 2A, 2B). *Ag-exp*CD4^+^ T cell effector function continued to deteriorate between days 9 and 15 of infection, with cells on day 15 of infection, expressing low levels of Ki67, ICOS, and CD25 and producing very low levels of TNF, IFN-γ, IL-2, and IL-10 (Fig. 2A, 2B, Supplemental Fig. 1A).

To assess mTOR activity in the *Ag-exp*CD4^+^ T cells as they became functionally exhausted during the course of *Py*NL infection, we examined the temporal expression of pS6, p4EBP1, pAKT^473^, and the metabolic regulator c-Myc. On day 5 of infection, when *Ag-exp*CD4^+^ T cells exhibited strong effector function, the cells expressed high levels of pS6, p4EBP1, pAKT^473^ and c-Myc (Fig. 2C, 2D). However, the expression of these molecules was not maintained, and in parallel with the loss of effector function, *Ag-exp*CD4^+^ T cells on day 15 of infection exhibited significantly reduced activation of pS6, p4EBP1, pAKT^473^, and c-Myc compared with *Ag-exp*CD4^+^ T cells on day 5 of infection (Fig. 2C, 2D). Importantly, detection of p4EBP1 in *Ag-exp*CD4^+^ T cells on days 5 and 15 of infection was almost completely blocked (similar to background staining within naive T cells) by treatment with the pan mTOR inhibitor Torin (Supplemental Fig. 1B). In contrast, the expression of pS6 was only reduced by ∼30% in *Ag-exp*CD4^+^ T cells on day 5 of infection following treatment with Torin, and expression was not modified in *Ag-exp*CD4^+^ T cells on day 15 of infection (Supplemental Fig. 1C). Thus, although pS6 and p4EBP1 are both frequently discussed as measures of mTORc1 activation and induction of anabolic metabolic programs (7–10), only p4EBP1 expression was Torin sensitive (and thus presumed to be mTOR dependent) in *Ag-exp*CD4^+^ T cells during malaria infection. mTOR-independent phosphorylation of S6^235/236^ in T cells has previously been shown (45). *Ag-exp*CD4^+^ T cells on day 15 of infection also showed a defect in capacity to engage the mTORc1 pathway following strong in vitro TCR stimulation compared with *Ag-exp*CD4^+^ T cells obtained from day 5 of infection, as determined by p4EBP1 expression (Supplemental Fig. 1D). Combined, these data demonstrate that CD4^+^ T cell functional exhaustion, as defined by lowered proliferation and reduced IFN-γ production, occurs alongside the apparent loss of mTORc1 activity during malaria infection.

### Exhausted CD4^+^ T cells have impaired nutrient uptake and exhibit loss of glycolytic cellular metabolism during malaria infection

**FIGURE 3.**
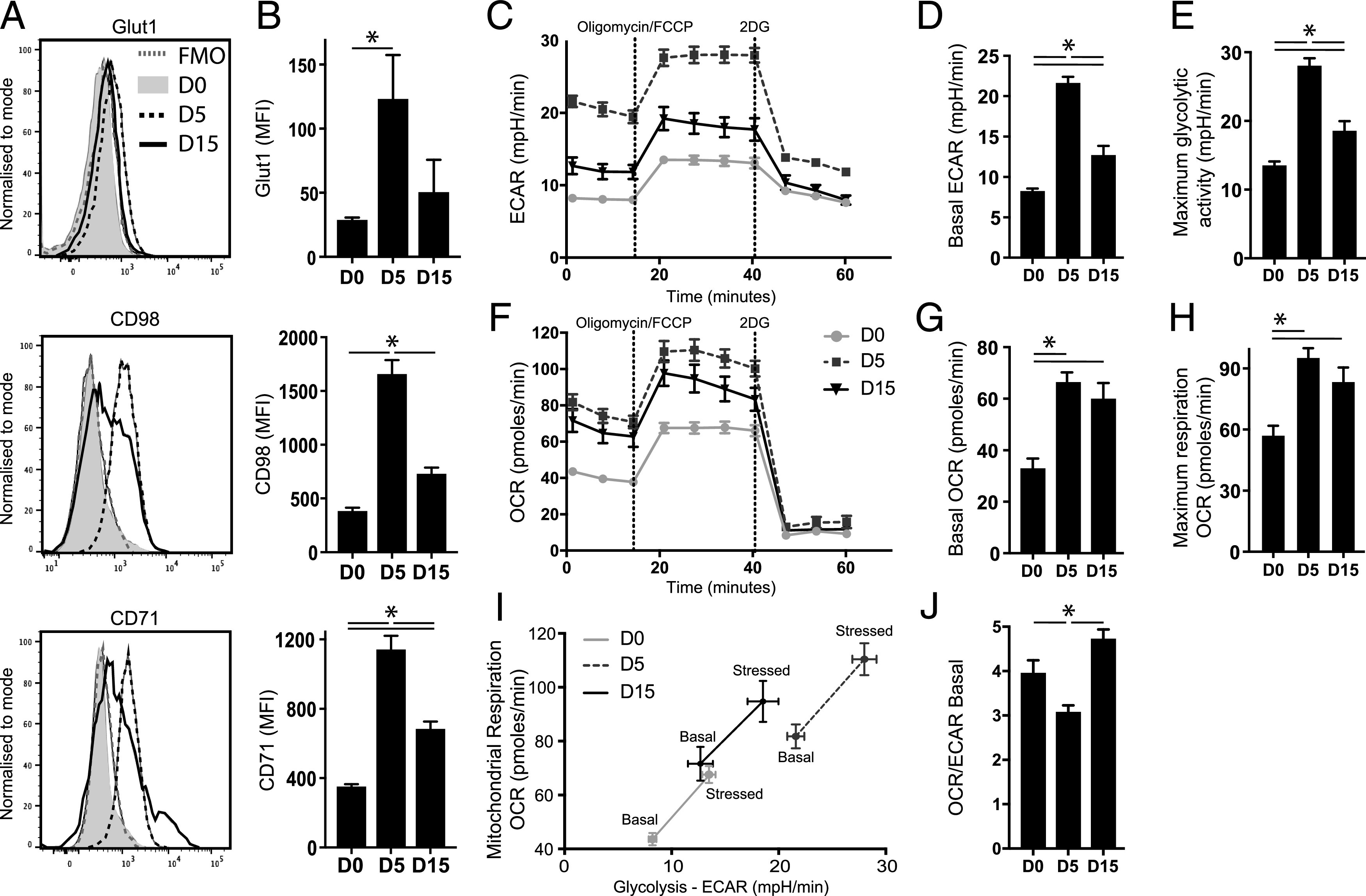
CD4^+^ T cells exhibit temporal loss in glycolytic capacity during the course of *Py*NL infection. C57BL/6 mice were infected i.v. with 1 × 10^4^ *Py*NL pRBCs. (**A**) Representative histograms and (**B**) calculated mean fluorescence intensity (MFI) (mean of the group) for Glut1, CD98, and CD71 in splenic *Ag-exp*CD4^+^ T cells during the course of infection, compared with naive CD4^+^ T cells from uninfected mice (day 0). (**C**–**E**) Seahorse analysis using modified mitochondrial stress test showing level of (C) ECAR and calculated (D) basal ECAR and (E) maximum glycolytic capacity of splenic CD4^+^ T cells from infected mice and naive mice. (**F**–**H**) Seahorse analysis showing level of (F) OCR and calculated (G) basal OCR and (H) maximal respiration of splenic CD4^+^ T cells from infected mice and naive mice. (**I**) Metabolic phenotype analysis and (**J**) the basal OCR:ECAR ratio of CD4^+^ T cells from naive and infected mice. Results are the mean ± SEM of the group (*n* = 4) and are from one of two or three independent experiments. *p < 0.05 by one-way ANOVA with Tukey post hoc test.

It is thought that mTOR has a major role in the integration of nutrient uptake and cellular metabolic pathways, including glycolysis (7–10). Consequently, we examined whether the reduction in mTORc1 activity in *Ag-exp*CD4^+^ T cells during *Py*NL infection translated to differences in glycolytic metabolism. On day 15 of infection, *Ag-exp*CD4^+^ T cells expressed lower levels of Glut-1 (a glucose transporter), CD98 (a component of the Slc7a5 heterodimeric large neutral amino acid transporter), and CD71 (a transferrin receptor) than *Ag-exp*CD4^+^ T cells from day 5 of infection (Fig. 3A, 3B). CD4^+^ T cells on day 15 of infection also exhibited a significantly lower basal and maximal ECAR (a measure of glycolysis) than CD4^+^ T cells from day 5 of infection, indicating that CD4^+^ T cells lost glycolytic capacity concurrent with onset of functional exhaustion during infection (Fig. 3C–E). Indeed, CD4^+^ T cells from day 15 of infection exhibited ECAR that was only modestly increased compared with resting CD4^+^ T cells from naive mice (Fig. 3C–E). However, in contrast with the dynamic alterations in ECAR, CD4^+^ T cells exhibited relatively stable basal and maximal OCR (a measure of mitochondrial respiration) during the course of infection, indicating that the CD4^+^ T cells maintained equivalent levels of oxidative phosphorylation during the course of *Py*NL infection (Fig. 3F–H). Thus, when analyses were combined, CD4^+^ T cells from day 15 of infection had a significantly diminished energetic profile and an increased OCR/ECAR metabolic ratio than cells from day 5 of infection (Fig. 3I, 3J).

### Reduction in glycolytic metabolism of *Ag-exp*CD4^+^ T cells during malaria infection correlates with loss of T-bet expression and the collapse of the metabolically active Th1 subset

**FIGURE 4.**
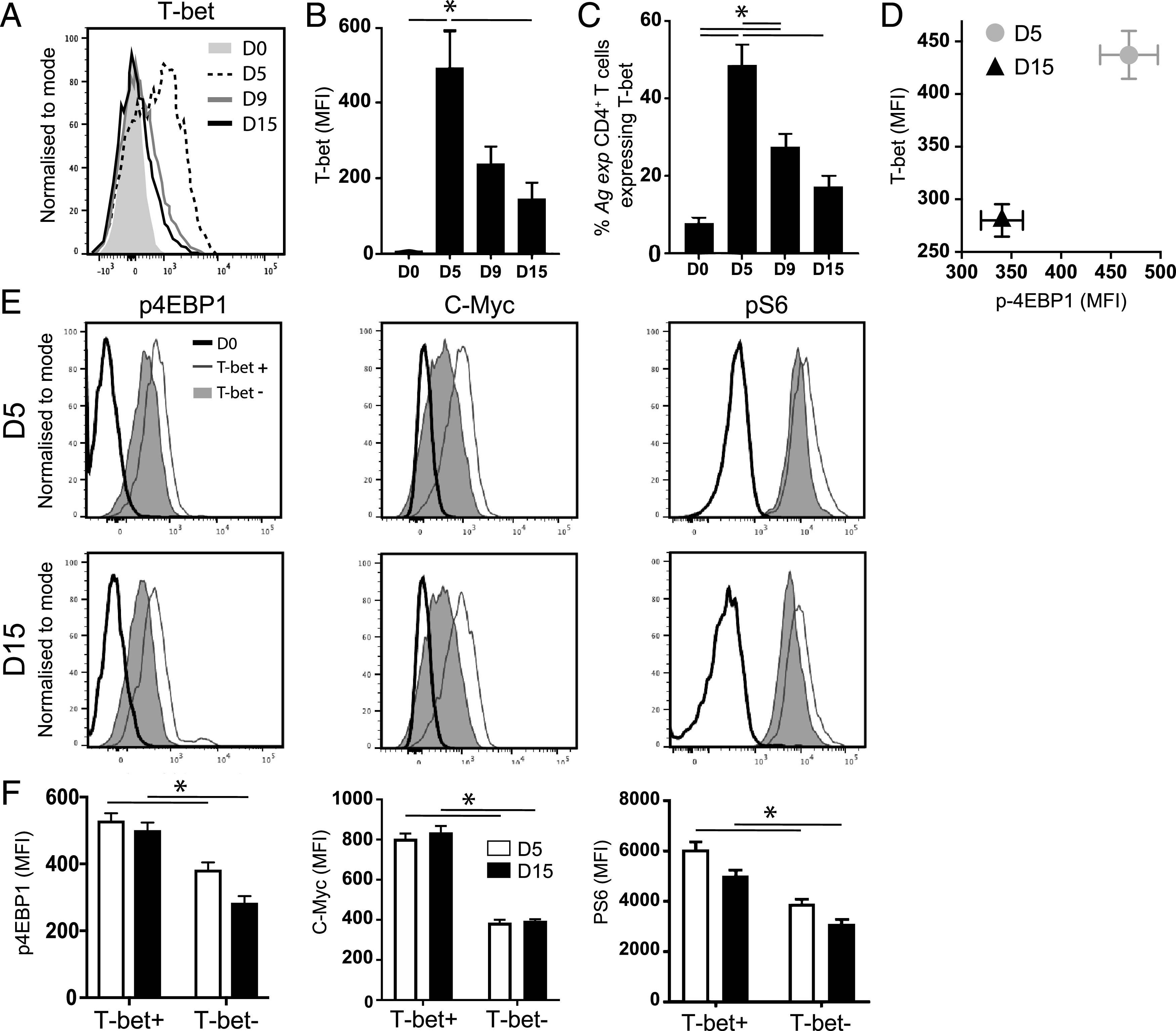
T cell exhaustion during *Py*NL infection is associated with loss of metabolically active T-bet^+^ cells. C57BL/6 mice were infected i.v. with 1 × 10^4^ *Py*NL pRBCs. (**A**) Representative histogram showing T-bet MFI within *Ag-exp*CD4^+^ T cells on days 5, 9, and 15 of infection compared with naive CD4^+^ T cells from uninfected mice. (**B**) Calculated mean fluorescence intensity (MFI) (mean of the group) of T-bet in *Ag-exp*CD4^+^ T cells and (**C**) the proportions of *Ag-exp*CD4^+^ T cells expressing T-bet on days 5, 9, and 15 of infection. (**D**) Correlation between T-bet and p4EBP1 levels in *Ag-exp*CD4^+^ T cells on days 5 and 15 of infection. (**E**) Representative histograms showing the MFI and (**F**) calculated MFI (mean of the group) of p4EBP1, c-Myc, and pS6 in gated splenic *Ag-exp*T-bet^+^ CD4^+^ T cells and *Ag-exp*T-bet^−^ CD4^+^ T cells on days 5 and 15 of infection compared with naive CD4^+^ T cells from uninfected mice. Results are the mean ± SEM of the group (*n* = 4) and are from one of two or three independent experiments. *p < 0.05 by one-way ANOVA with Tukey post hoc test or Mann–Whitney *U* test.

The above data are in agreement with recent studies suggesting that T cell exhaustion is associated with loss of mTOR activity and metabolic dysfunction (12, 46, 47). We consequently examined the molecular basis for the reduction in mTOR activity in exhausted T cells during malaria infection. We observed a dramatic reduction in T-bet expression by *Ag-exp*CD4^+^ T cells and a reduced proportion of *Ag-exp*CD4^+^ T cells expressing T-bet (Th1 cells) as *Py*NL infection progressed and T cell exhaustion was established (Fig. 4A–C). T-bet expression was directly correlated with p4EBP1 expression in *Ag-exp*CD4^+^ T cells during infection (Fig. 4D). In agreement, *Ag-exp*T-bet^+^ Th1 cells exhibited higher and sustained mTOR activity, as indicated by p4EBP1 expression [the expression of which, above baseline levels observed in naive T cells, was Torin sensitive (Supplemental Fig. 1E)] and an elevated metabolic signature (c-Myc and pS6) compared with *Ag-exp*T-bet^−^ non-Th1 cells on both days 5 and 15 of infection (Fig. 4E, 4F, Supplemental Fig. 2A). Although *Ag-exp*T-bet^−^ T cells were a heterogeneous population, they were predominantly CD44^+^CD62L^−^ Foxp3^−^ Gata-3^−^, non-Tfh (CXCR5^−^PD-1^−^) effector T cells (Supplemental Fig. 2B, 2C). Exhausted CD4^+^ T cells have previously been shown to exhibit an equivalent nonpolarized phenotype during lymphocytic choriomeningitis virus infection (48, 49).

**FIGURE 5.**
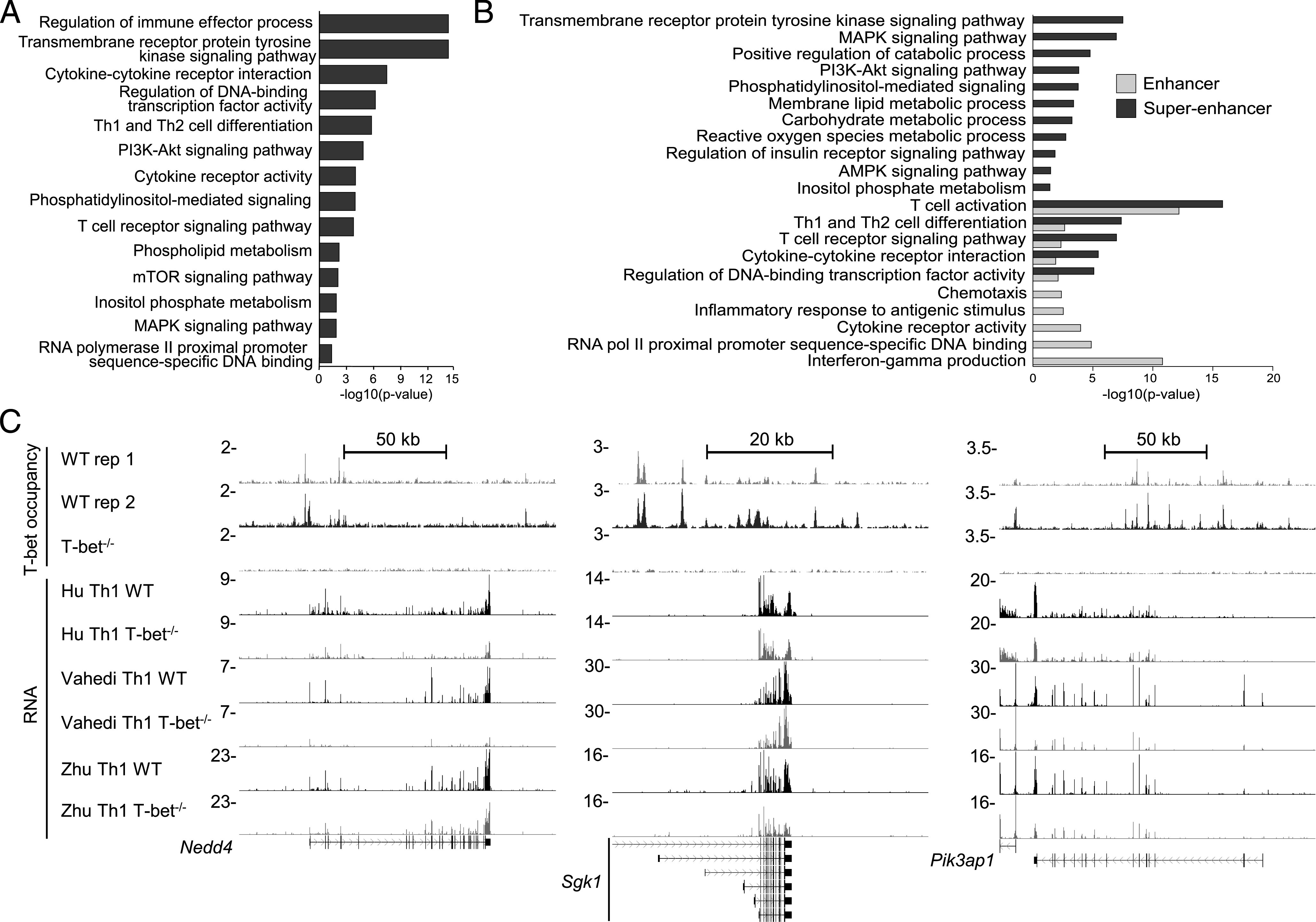
T-bet activates expression of genes of the mTOR signaling pathway and signal transduction cascades that regulate mTOR function. (**A**) Enrichment, shown with a −log_10_ *p* value, of representative Gene Ontology (GO) biological process, GO molecular function, and KEGG pathways in the set of genes significantly downregulated in T-bet^−/−^ versus WT CD4 T cells polarized under Th1 conditions. (**B**) Enrichment, shown with a −log_10_ *p* value, of representative GO biological process, GO molecular function, and KEGG pathways in the set of genes bound by T-bet at a typical enhancer or bound by T-bet at a superenhancer. (**C**) T-bet occupancy (two replicates) and RNA abundance [data from three studies (34–36)] at selected genes in the mTOR signaling pathway in WT and T-bet^−/−^ cells.

These data raised the question whether the temporal reduction of mTORc1 activity (as inferred by lower p4EBP1 expression) and glycolytic metabolism in the exhausted *Ag-exp*CD4^+^ T cell population during malaria infection was a consequence of the loss of T-bet. To test this, we performed a meta-analysis of published RNA-seq data (34–36) from T-bet–null versus WT CD4^+^ T cells polarized under Th1 conditions in vitro to identify genes downregulated in effector CD4^+^ T cells upon loss of T-bet expression (Supplemental Fig. 3A). This revealed enrichment of genes with functions in Gene Ontology categories. such as regulation of immune effector process, and Kyoto Encyclopedia of Genes and Genomes (KEGG) pathways, such as Th1 and Th2 cell differentiation, as might be expected, but also downregulation of genes in the mTOR signaling pathway and the PI3K–AKT and MAPK signaling pathways that regulate mTOR function (Fig. 5A). To address whether T-bet directly regulated these genes, we identified the enhancers and superenhancers bound by T-bet in Th1 cells. As we have found previously (41), genes associated with T-bet–bound superenhancers had functions related to IFN-γ production, inflammatory response, and chemotaxis (Fig. 5B). In contrast, genes associated with T-bet–bound typical enhancers were enriched in functions relating to metabolism and PI3K–AKT MAPK, AMPK, and insulin receptor signaling (Fig. 5B). Thus, T-bet regulates metabolic functions through a distinct set of elements to those used to regulate the inflammatory response. Genes bound and activated by T-bet included *Sgk1*, *Nedd4*, and *Pik3ap1* (Fig. 5C), which function in pathways that positively regulate mTOR activity (50–53). These results suggest that T-bet may control mTOR activity in effector T cells and that loss of T-bet may, in part, contribute to loss of mTOR activity and glycolytic metabolism within T cells during establishment of cellular exhaustion.

### mTOR does not sustain T-bet expression and is necessary but not sufficient for IFN-γ production in established Th1 effector cells

**FIGURE 6.**
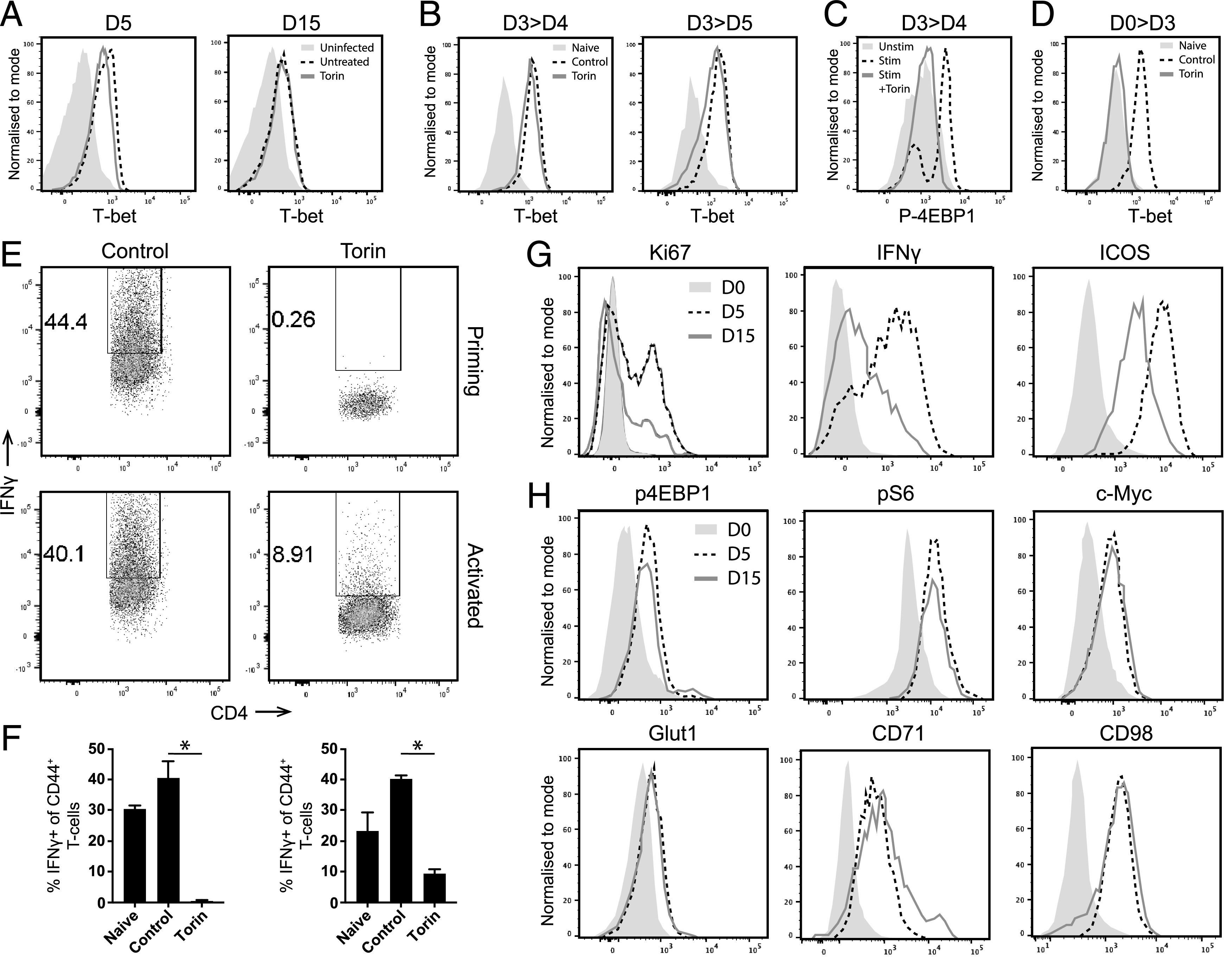
mTOR does not actively control T-bet expression and is necessary but not sufficient for IFN-γ production in established effector Th1 cells. (**A**) Representative histograms showing T-bet mean fluorescence intensity (MFI) in splenic *Ag-exp*CD4^+^ T cells obtained from days 5 and 15 of *Py*NL infection incubated ex vivo with Torin or control for 4 h and compared with naive CD4^+^ T cells from uninfected mice (day 0). (**B**–**F**) CD4^+^ T cells from C57BL/6 mice were stimulated in vitro with anti-CD3 and anti-CD28 under Th1 conditions in presence of Torin (or control) added on either day 0 or 3 poststimulation. (B and C) Representative histograms showing (B) T-bet and (C) p4EBP1 MFI in Th1-differentiated CD4^+^ T cells when Torin or control was administered on day 3, with analysis on day 4 or 5, compared with naive unstimulated CD4^+^ T cells. (D) Representative histogram showing T-bet MFI in Th1-differentiated CD4^+^ T cells when Torin or control was administered on day 0, with analysis on day 3, compared with naive unstimulated CD4^+^ T cells. (E) Representative dot plots showing IFN-γ levels in Th1-differentiated CD4^+^ T cells when Torin was administered on day 0 (at priming) or on day 3 of stimulation (activated), with analysis on day 4 of stimulation. (F) The proportion of CD4^+^CD44^+^ T cells producing IFN-γ when stimulated under Th1 conditions when Torin was added at priming (left graph) or on day 3 of activation (right graph), with analysis on day 4, compared with unstimulated CD4^+^ T cells. (**G**) Representative histograms showing Ki67, IFN-γ, and ICOS MFI by *Ag-exp*T-bet^+^ CD4^+^ T cells on days 5 and 15 of infection, compared with naive CD4^+^ T cells from uninfected mice (day 0). (**H**) Representative histograms showing the MFI of metabolism-related molecules on gated *Ag-exp*T-bet^+^ Th1 cells on days 5 and 15 of infection, compared naive CD4^+^ T cells from uninfected mice (day 0). Results are the mean ± SEM of the group (F), technical replicates (*n* = 3). The results are representative of two separate experiments with (A, G, and H) (*n* = 4 per group). *p < 0.05 by one-way ANOVA with Tukey post hoc test.

Although the above results suggested that T-bet may positively effect mTOR activity, it has also previously been shown that mTOR promotes T-bet activity in T cells (54–56), suggesting that T-bet and mTOR may exist in an interlinked pathway to coordinate lineage commitment and cellular metabolic programming within effector T cells. Consequently, to examine whether a reduction in mTOR activity caused lowered T-bet expression in exhausted *Ag-exp*CD4^+^ T cells during malaria infection, we treated *Ag-exp*CD4^+^ T cells obtained from mice on days 5 and 15 of *Py*NL infection with Torin. Interestingly, Torin failed to modify expression of T-bet in *Ag-exp*CD4^+^ T cells obtained on either day of infection (Fig. 6A). Moreover, treatment of in vitro–generated Th1 cells with Torin on day 3 of activation did not modify T-bet expression or alter T-bet nuclear translocation when the Th1 cells were examined on days 4 or 5 of stimulation (Fig. 6B, Supplemental Fig. 3B). This was evident, even though Torin inhibited p4EBP1 expression (to the background staining level observed in unstimulated naive T cells), showing it was likely effective at inhibiting mTORC1 in Th1 cells (Fig. 6C). In contrast, administration of Torin at the initiation of in vitro CD4^+^ T cell activation and Th1 differentiation completely abrogated T-bet expression (Fig. 6D). Thus, these data suggest that, although mTOR is necessary for induction of T-bet during T cell priming, it is not required for sustained T-bet expression in activated Th1 cells during infection. As such, the reduction in T-bet expression in exhausted *Ag-exp*CD4^+^ T cells during malaria infection may not be directly due to the inhibition of mTOR activity.

In contrast to the effects on T-bet, we observed that Torin administration inhibited IFN-γ production by CD4^+^ T cells during in vitro priming as well as by in vitro–activated and fully differentiated Th1 cells (Fig. 6E, 6F). This shows that mTOR activity is necessary for IFN-γ production by Th1 cells. Nevertheless, *Ag-exp*T-bet^+^ Th1 cells on day 15 of infection exhibited reduced proliferation (Ki67^+^), function (IFN-γ^+^), and activation (ICOS^+^) compared with *Ag-exp*T-bet^+^ Th1 cells on day 5 of infection (Fig. 6G). This was despite the *Ag-exp*T-bet^+^ Th1 cells from days 5 and 15 of *Py*NL infection expressing equivalent levels of p4EBP1, pS6, pAKT^473^, and c-Myc (Fig. 6H). Consequently, these results imply that mTOR activity is not sufficient to maintain Th1 cell effector function during *Py*NL infection, with Th1 cell function (as defined by Ki67 expression and IFN-γ production) restrained during malaria infection in the absence of alterations in mTOR activity and nutrient transporter expression.

### PD-1 and CTLA-4 control of CD4^+^ T cell glycolytic metabolism is associated with control of T-bet expression, but they do not influence mTOR activity within mature Th1 cells during *Py*NL infection

**FIGURE 7.**
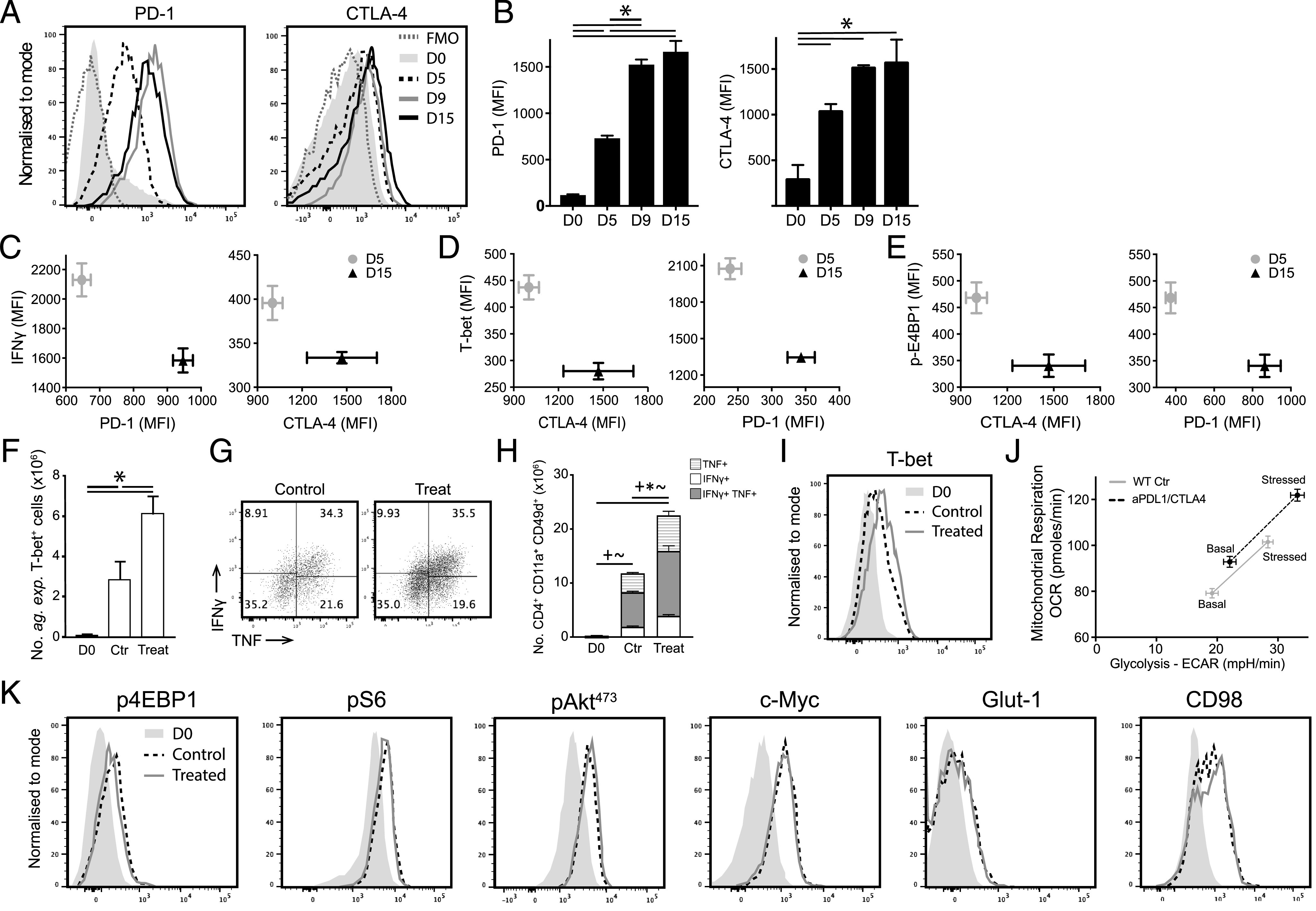
PD-1 and CTLA-4 control T-bet expression and glycolytic metabolism of total CD4^+^ T cells but do not influence the glycolytic metabolic signature of mature Th1 cells during malaria infection. C57BL/6 mice were infected i.v. with 1 × 10^4^ *Py*NL pRBCs and were administered i.p with anti–PDL-1 and anti–CTLA-4 or control every second day from day 5 postinfection. (**A**) Representative histograms and (**B**) calculated mean fluorescence intensity (MFI) (mean of the group) of PD-1 and CTLA-4 in *Ag-exp* CD4^+^ T cells during the course of infection compared with naive CD4^+^ T cells from uninfected mice (day 0). (**C**–**E**) Correlation between PD-1 and CTLA-4 with (C) IFN-γ, (D) T-bet, and (E) p4EBP1 in *Ag-exp*CD4^+^ T cells on days 5 and 15 of infection. (**F**) Numbers of *Ag-exp*T-bet^+^ CD4^+^ T cells on day 15 postinfection in anti–PDL-1– and anti–CTLA-4–treated and control-treated mice compared with naive CD4^+^ T cells from uninfected mice (day 0). (**G**) Representative dot plot showing IFN-γ and TNF levels by *Ag-exp*T-bet^+^ CD4^+^ T cells and (**H**) the numbers of IFN-γ and/or TNF-positive *Ag-exp*CD4^+^ T cells on day 15 postinfection. (**I**) Representative histogram showing T-bet MFI in splenic CD4^+^ T cells from anti–PD-1­– and anti–CTLA-4–treated and control-treated mice on day 15 of infection. (**J**) Seahorse metabolic phenotype analysis of splenic CD4^+^ T cells from anti–PDL-1– and anti–CTLA-4–treated and control-treated mice on day 15 of infection. (**K**) Representative histograms showing the MFI of metabolism-related molecules by splenic *Ag-exp*T-bet^+^ Th1 cells from anti–PDL-1– and anti–CTLA-4–treated and control-treated mice on day 15 of infection, compared with naive CD4^+^ T cells from uninfected mice (day 0). Results are the mean ± SEM of the group (*n* = 4 or 5) and are from one of three independent experiments. *p < 0.05 between defined groups with (H), *p < 0.05 for differences in IFN-γ^+^ cell numbers, ∼*p* < 0.05 for differences in IFN-γ^+^TNF^+^ cell numbers, +*p* < 0.05 for differences in TNF^+^ cell numbers by one-way or two-way ANOVA with Tukey post hoc test.

As mTOR did not appear to directly control T-bet expression in *Ag-exp*CD4^+^ T cells during malaria infection, we questioned which other pathways may influence T-bet to affect *Ag-exp*CD4^+^ T cell glycolytic metabolism and cellular exhaustion during *Py*NL infection. The regulatory pathways PD-1 and CTLA-4 have been shown to suppress T cell function during malaria infection and to influence cellular metabolism in other models (15, 18–20, 23, 28, 29). As expected, the expression of PD-1 and CTLA-4 was upregulated on *Ag-exp*CD4^+^ T cells during the course of malaria infection (Fig. 7A, 7B) and was inversely correlated with IFN-γ production (Fig. 7C). Notably, the expression of PD-1 and CTLA-4 was also inversely correlated with both T-bet and p4EBP1 expression during malaria infection (Fig. 7D, 7E). Consistent with this, anti–PD-L1 and anti–CTLA-4 administration to block T cell exhaustion (Fig. 7F–H) led to an increase in T-bet expression within the overall CD4^+^ T cell population (Fig. 7I) and corresponded with a significant increase in the level of cellular glycolysis and oxidative phosphorylation by CD4^+^ T cells on day 15 of infection (Fig. 7J). Similarly, IL-27 has been identified as a major controller of T cell exhaustion, including during malaria infection (25, 26, 57) (Supplemental Fig. 4A–C), and CD4^+^ T cells from infected WSX-1^−/−^ mice exhibited higher T-bet expression than CD4^+^ T cells from infected WT mice (on day 13 postinfection), which corresponded with significantly higher levels of glycolysis (Supplemental Fig. 4D, 4E). Combined, these results support the conclusion that regulatory pathways influence the metabolic status of the effector CD4^+^ T cell population during malaria infection by impacting T-bet expression and the proportion of Th1 cells.

Interestingly, anti–PDL-1 and anti–CTLA-4 treatment did not affect the level of mTORc1 activity (as inferred from p4EBP1 expression) or metabolic programming of *Ag-exp*Th1 cells on a cell-per-cell basis compared with *Ag-exp*Th1 cells from control-treated–infected mice (Fig. 7K). Moreover, despite their hyperactivated functional state (Supplemental Fig. 4B, 4C), *Ag-exp*T-bet^+^ Th1 cells from infected WSX-1^−/−^ mice did not exhibit increased expression of mTORc1-associated molecules or nutrient transporters compared with *Ag-exp*T-bet^+^ Th1 cells from infected WT mice (Supplemental Fig. 4F). Consequently, our results indicate that regulatory pathways do not appear to directly affect mature Th1 cell metabolism and further support the hypothesis that Th1 cell activity can be modulated in vivo during infection in the absence of differences in expression and activation of mTORc1 and glycolysis-associated molecules within mature Th1 cells.

## Discussion

We have shown that mTOR activity and glycolytic metabolic programming was not sustained in the *Ag-exp*CD4^+^ T cell population throughout the course of *Py*NL infection, with repression of mTOR activity and cellular glycolysis corresponding with the temporal suppression of *Ag-exp*CD4^+^ T cell functionality. Although the phenomenon of T cell exhaustion during malaria infection has previously been characterized (15–17), to our knowledge, this is the first report to show that it occurs concomitant with loss of glycolytic metabolism and impaired mTOR activity within the dysregulated T cells.

The downregulation of mTORc1 activity in exhausted *Ag-exp*CD4^+^ T cells during the course of malaria infection, along with reduction in cellular glycolysis, was comparable to that observed for exhausted Ag-specific CD8^+^ T cells during chronic viral infections (12, 46). Therefore, these results support a model in which effector T cell exhaustion is propagated by metabolic insufficiency due to restricted uptake of nutrients (47). This could be further enforced by the metabolic derangement, including hypoglycemia, that is frequently associated with blood-stage malaria (58, 59). However, an important observation from our phenotypic analyses was that the onset of *Ag-exp*CD4^+^ T cell exhaustion was characterized by a significant restructuring of the *Ag-exp*CD4^+^ T cell compartment, in particular a dramatic decline in T-bet expressing (Th1) cells. As the dwindling *Ag-exp*T-bet^+^ Th1 cells exhibited higher mTOR activity and nutrient transporter expression than *Ag-exp*T-bet^−^ T cells throughout the course of infection, these data are suggestive that T-bet may be a major controller of metabolic programming and mTOR activation in *Ag-exp*CD4^+^ T cells during malaria infection.

This hypothesis is supported by our observation that lack of T-bet led to reduction in the expression of genes within effector CD4^+^ T cells in pathways related to cellular metabolism and activation of the mTOR signaling pathway, including Sgk1, which inhibits TSC2 (50), Nedd4, which inhibits PTEN (51, 52), and Pik3ap1, which influences AKT and Pi3K (53). Interestingly, T-bet–mediated regulation of these genes was through a distinct binding profile compared with genes encoding inflammatory mediators, which are regulated by T-bet through superenhancers (41, 60). In addition, it has also previously been shown that T-bet can influence the metabolic state of *Ag-exp*CD4^+^ T cells indirectly through binding and inhibiting the transcriptional repressor BCL-6, which regulates a number of glycolysis-related genes (61). CD8^+^ T cell exhaustion has also been associated with loss of T-bet expression in other conditions (62), and T-bet^low^Eomes^hi^ exhausted CD8^+^ T cells exhibit greater metabolic dysfunction than T-bet^hi^Eomes^low^ CD8^+^ T cells (46, 47). Thus, whereas additional work temporally manipulating T-bet expression in effector T cells during the course of malaria infection is required to confirm our conclusion, it is possible that loss of T-bet corresponds with lowered mTOR activity and glycolytic metabolism in exhausted CD4^+^ T cells during chronic conditions.

The role of mTOR in influencing T-bet expression and activity in effector CD4^+^ T cells during malaria infection is unclear. We have shown, in agreement with previous studies (54, 55), that mTOR is required to promote T-bet expression during the initial activation of T cells. However, as in a recent proteomics study from the Cantrell group (63), mTOR activity did not appear to be critical, at least in the short-term, to sustain T-bet protein levels or nuclear translocation in effector Th1 cells, indicating that mTOR has distinct temporal roles in coordinating various cellular processes within newly activated T cells compared with mature effector T cells. Nevertheless, directly blocking mTOR activity attenuated IFN-γ production by T cells during activation, as well as by mature effector Th1 cells. Although this is consistent with observations that T cell effector function may be restricted when uptake of nutrients (such as glucose) and generation of glycolytic metabolites falls below a threshold (13, 14), our results also showed that *Ag-exp*T-bet^+^ Th1 cells exhibited a substantial loss of effector function at the later stages of malaria despite sustaining mTOR pathway activity. Thus, overall, our data argue that mTOR is necessary but not sufficient for sustaining T cell effector activity during an immune response with additional mechanisms uncoupled or downstream from mTOR (and potentially glycolysis) that allow effector function to be regulated in activated Th1 cells. Further work is required to fully define the effects of the mTOR pathway on T-bet activity and on the function of effector CD4^+^ T cells during infection.

In terms of other pathways that orchestrate CD4^+^ T cell exhaustion during malaria infection and how this is related to regulation of T-bet expression and T cell metabolism, we focused on the roles of PD-1, CTLA-4, and IL-27. In agreement with previous studies (18, 20, 23, 25, 26), we confirmed through combinatorial blockade of PD-1 and CTLA-4 and through using IL-27R^−/−^ mice that the pathways regulate the magnitude of the *Ag-exp*CD4^+^ T cell and *Ag-exp*T-bet^+^ Th1 cell responses during malaria infection. Blockade of PD-1 and CTLA-4 and abrogation of IL-27R signaling thus raised the level of T-bet within the overall CD4^+^ T cell population, and this directly correlated with an increase in CD4^+^ T cell glycolytic metabolism during infection. However, inhibition of these pathways did not modify the level of mTOR activity as measured by p4EBP1 expression or the glycolytic programming of Th1 cells. Whether these pathways influenced mitochondrial mass and polarization [which is modified in exhausted CD8^+^ T cells (46)], or other metabolic pathways, such as amino acid metabolism, to influence Th1 effector function during infection will require additional investigation.

That anti–PD-1 and anti–CTLA-4 treatment did not affect mTOR activity in differentiated *Ag-exp*Th1 cells during malaria infection but did raise the level of T-bet and glycolysis in total CD4^+^ T cells is consistent with a model in which combinatorial checkpoint inhibitor treatment (re)invigorated the activation of non-Th1 precursor exhausted CD4^+^ T cells. This likely facilitated the proliferation and (re)differentiation of the precursor cells into highly glycolytic and metabolically active Th1 cells. Indeed, intermediate activated CD8^+^ T cell stem cells, rather than terminally differentiated and exhausted effector CD8^+^ T cells, have been shown to provide the proliferative burst after checkpoint immunotherapy during chronic viral infection (64). Why PD-1 and CTLA-4 may influence mTOR activity in immature or stem cells rather than mature effector T cells may relate to their influence on the CD28 pathway. CD28 promotes metabolic reprogramming and mTOR activation during T cell activation (9, 65), and the capacity of PD-1 and CTLA-4 to inhibit mTOR pathways and glycolysis in CD4^+^ T cells during in vitro priming (28, 29) is likely linked to antagonism of CD28 signaling (66, 67). Mature effector Th1 cells depend less on CD28 costimulation than do naive T cells (68), and interestingly, the rescue of exhausted CD8^+^ T cells following anti–PD-1 treatment depends upon CD28 costimulation (69).

In summary, we have shown that the functional exhaustion of the CD4^+^ T cell response during malaria infection occurs concomitantly with alterations in mTOR activation and reduced metabolic fitness. We have demonstrated that this decline in glycolytic metabolism in exhausted *Ag-exp*CD4^+^ T cells is related to the loss of T-bet expression, with *Ag-exp*Th1 cells maintaining high levels of mTOR activity and glycolytic metabolism during infection. How mTOR activity is regulated in *Ag-exp*Th1 cells and its overall importance for maintaining Th1 lineage specification and cellular function requires additional investigation, but direct dysregulation of mTOR activity may not be the principal reason for collapse of the Th1 response during malaria infection. These results have important implications for the design of immunotherapies to reactivate Ag-specific CD4^+^ T cell responses during infection and cancer.

## Supplementary Material

Data Supplement
