# Supplementary material for: Exhausted CD4+ T Cells during Malaria Exhibit Reduced mTORc1 Activity Correlated with Loss of T-bet Expression
Source: J Immunol. 2020 Aug 17;205(6):1608–19. doi: 10.4049/jimmunol.2000450 (PMC7477746; doi:10.4049/jimmunol.2000450)
Supplement: Data Supplement [file JI_2000450.zip › JI_2000450_Supplemental_Figures_1.pdf]

Supplemental Figure 1

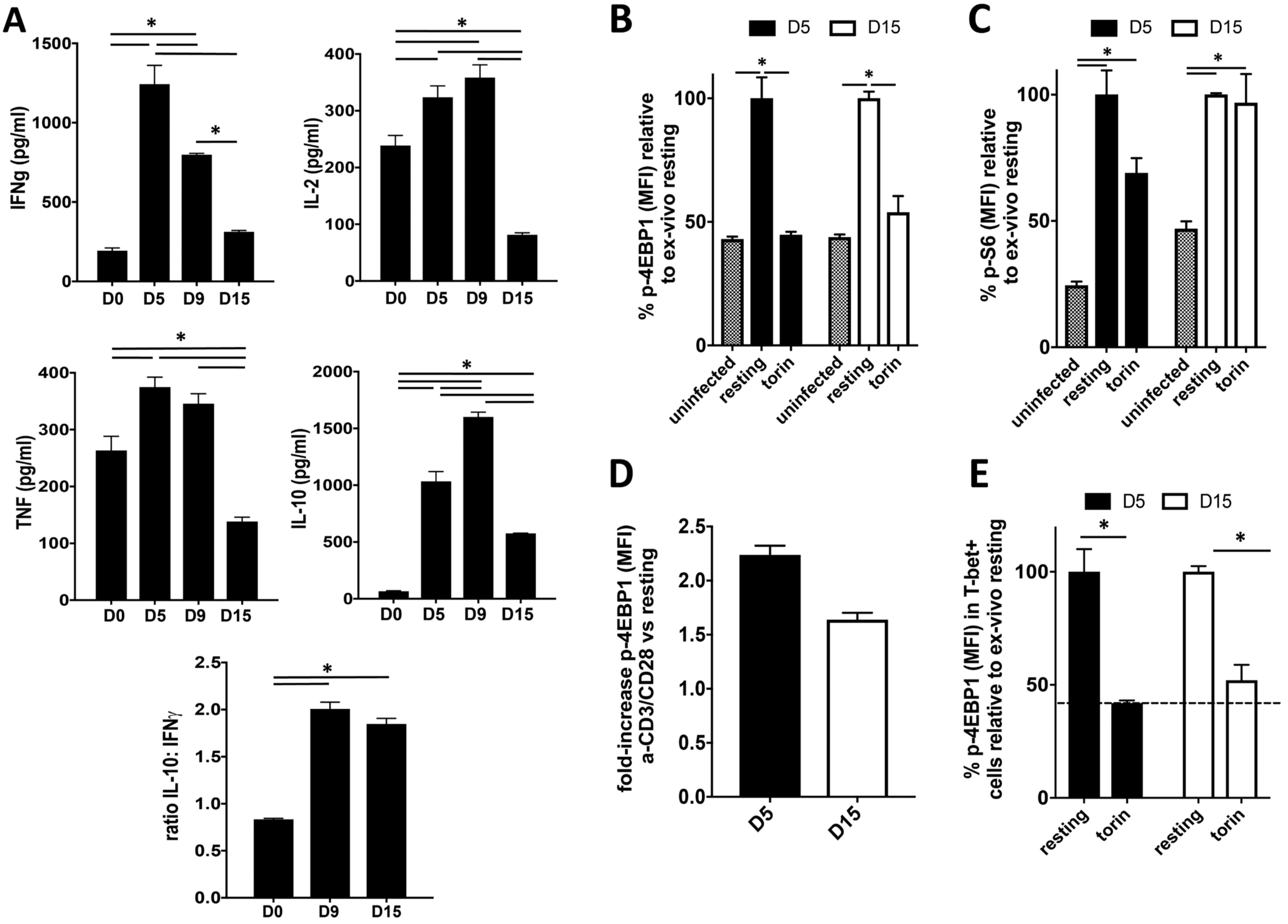

S1 Fig: CD4$^+$ T cells lose capacity to produce cytokines during the course of infection and p4EBP1 expression in *Ag-exp*CD4$^+$ T cells is mTOR-inhibitor Torin-sensitive

(A) The level of cytokines in the supernatant of purified CD4$^+$ T cells from naïve and *Py*NL infected mice after stimulation *in vitro* for 4 h with PMA/ionomycin, measured by cytokine bead array. Results are the mean ± SEM of the group (n = 4 or 5) and are from one of two independent experiments. *, $P < 0.05$ between defined groups (1-way ANOVA with Tukey's *post hoc* test). **(B-E)** Splenocytes from days 5 and 15 of *Py*NL infection, and from uninfected mice, were incubated *ex vivo* with Torin or vehicle for 4 h. (**B**) p4EBP1 and (**C**) pS6 MFI in Torin treated *Ag-exp*CD4$^+$ T cells relative to control (vehicle treated, resting) *ex vivo* infection-derived cells. The MFI of molecules in naïve CD4$^+$ T cells from uninfected mice is inferred to represent background staining. (**D**) The fold increase in p4EBP1 MFI in splenic *Ag-exp*CD4$^+$ T cells from days 5 and 15 infection stimulated for 40 min with anti-CD3 and anti-CD28 (presented relative to MFI in resting non-stimulated *Ag-exp*CD4$^+$ T cells from respective day of infection). (**E**) p4EBP1 MFI in *Ag-exp*T-bet$^+$ T cells treated with Torin relative to expression in control (vehicle treated, resting) *ex vivo* infection-derived cells (dotted line represents baseline p-4EBP1 MFI in naïve CD4$^+$ T cells from uninfected mice). Results are the mean ± SEM of the group (n = 3) and are from one of three independent experiments. *, $P < 0.05$ between defined groups (2-way ANOVA with Tukey's *post hoc* test and t-test).

Supplemental Figure 2

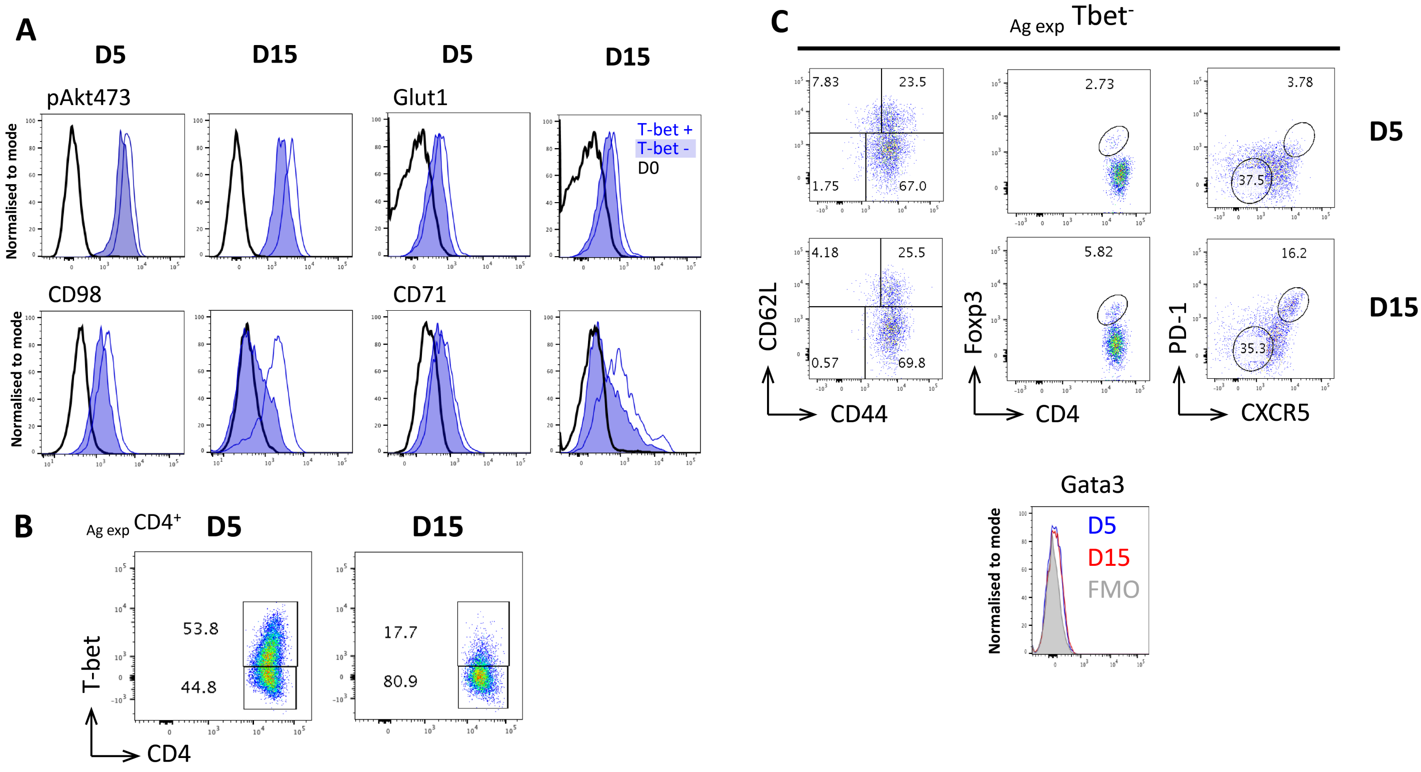

S2 Fig. The phenotype and metabolism of *Ag-exp*T-bet$^-$ CD4$^+$ T cells during infection.

C57BL/6 mice were infected i.v. with 10^4 *Py*NL pRBCs. (**A**) Representative histograms showing the expression of metabolism-related molecules by splenic *Ag-exp*T-bet$^+$ CD4$^+$ T cells and *Ag-exp*T-bet$^-$ CD4$^+$ T cells on day 5 and day 15 of infection. (**B**) Representative dot plots showing the gating of splenic T-bet$^+$ and T-bet$^-$ *Ag-exp*CD4$^+$ T cells on day 5 and day 15 of infection. (**C**) Representative dot plots showing (left) CD44 and CD62L expression (middle) Foxp3 expression and (right) CXCR5 and PD-1 (bottom) GATA3 expression by *Ag-exp*T-bet$^-$ CD4$^+$ T cells on day 5 and day 15 of infection. Results represent the mean of the group (n = 4 or 5) and are from one of three independent experiments.

Supplemental Figure 3

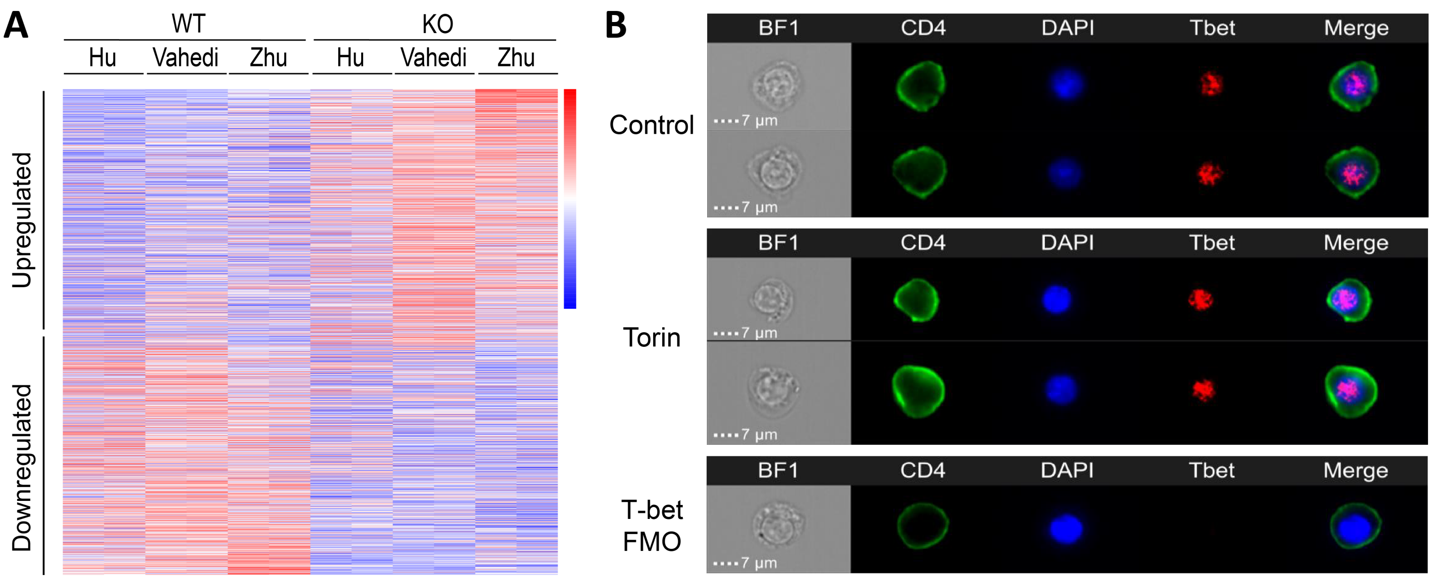

S3 Fig. Identification of genes consistently upregulated and downregulated in T-bet$^{-/-}$ Th1 cells and influence of mTOR on T-bet nuclear translocation in Th1 cells.

(A) Heatmap of genes up and downregulated (padj<0.01) in T-bet$^{-/-}$ versus WT CD4 T cells polarized under Th1 conditions. Data are taken from three studies (**34-36**), with two replicates per genotype in each study. Log2 fold-change in expression (T-bet$^{-/-}$ / WT) is colored according to the scale on the right. **(B)** ImageStream analysis of T-bet levels in *in vitro* generated Th1 cells when Torin was administered for 24 h on day 3 post-stimulation, with analysis on day 4 of stimulation. The results are representative of 2 separate experiments.

Supplemental Figure 4

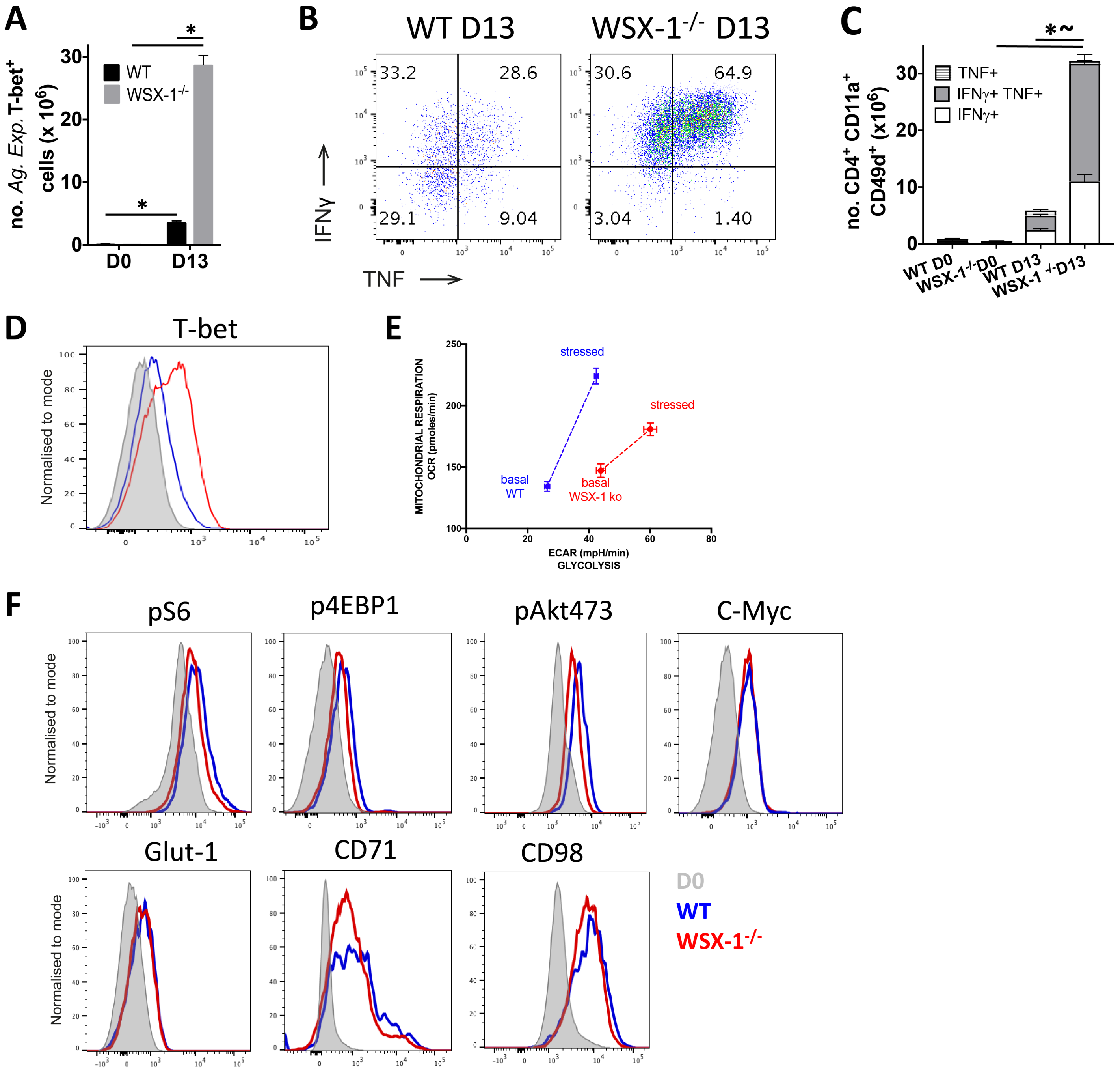

S4 Fig: IL-27 controls T-bet expression and glycolytic metabolism of total CD4+ T cells but does not influence the glycolytic metabolic signature of mature Th1 cells during malaria.

C57BL/6 and IL-27R$^{-/-}$ (WSX-1$^{-/-}$) mice were infected i.v. with 10^4 *P. berghei* NK65 pRBCs. **(A)** Numbers of *Ag-exp*CD4$^+$ T cells on day 13 of infection. **(B)** Representative dot plots showing IFN-**γ** and TNF levels and **(C)** the numbers of IFN- **γ** and /or TNF positive *Ag-exp*CD4$^+$ T cells on day 13 post-infection. (**D)** T-bet MFI in splenic CD4$^+$ T cells from WSX-1$^{-/-}$ and WT mice on day 13 of infection. **(E)** Seahorse metabolic phenotype analysis of splenic CD4$^+$ T cells from WSX-1$^{-/-}$ and WT mice on day 13 of infection. **(F)** Representative histograms showing the MFI of metabolism-related molecules by splenic *Ag-exp*T-bet$^+$ Th1 cells from WT and WSX-1$^{-/-}$ mice on day 13 of infection. Results are the mean ± SEM of the group (n = 4 or 5) and are from one of two-three independent experiments. *, $P< 0.05$ between defined groups, with (**C**) * $P< 0.05$ for differences in IFN-γ$^+$ cell numbers and ~ $P< 0.05$ for differences in IFN-γ$^+$TNF$^+$ cell numbers (2-way ANOVA with Tukey's *post hoc* test).
